# Dexmedetomidine to reduce vasopressor resistance in refractory septic shock: Protocol for a double-blind randomized controlled pilot trial (ADRESS Pilot study)

**DOI:** 10.3389/fmed.2022.968274

**Published:** 2022-08-09

**Authors:** Auguste Dargent, Abderrahmane Bourredjem, Laurent Argaud, Bruno Levy, Isabelle Fournel, Amélie Cransac, Julio Badie, Luc Quintin, Jean-Pierre Quenot

**Affiliations:** ^1^Hospices Civils de Lyon, Hôpital Edouard Herriot, Service de Médecine Intensive-Réanimation, Lyon, France; ^2^APCSe VetAgro Sup UPSP 2016.A101, Marcy l'Etoile, France; ^3^INSERM, CIC 1432, Module Epidémiologie Clinique, Dijon, France; ^4^CHU Dijon-Bourgogne, Centre d'Investigation Clinique, Épidémiologie Clinique/essais Cliniques, Dijon, France; ^5^Université de Lyon, Université Claude Bernard Lyon 1, Faculté de médecine Lyon-Est, Lyon, France; ^6^Service de Réanimation Médicale, Centre Hospitalier Universitaire Nancy Brabois, Nancy, France; ^7^Institut du Cœur et des Vaisseaux, Groupe Choc, équipe 2, Inserm U1116, Faculté de Médecine, Nancy-Brabois, France; ^8^Department of Pharmacy, Dijon University Hospital, Dijon, France; ^9^LNC-UMR1231, University of Burgundy and Franche-Comté, Dijon, France; ^10^Hôpital Nord Franche-Comté, Service de Médecine Intensive-Réanimation, Trévenans, France; ^11^Hôpital d'instruction des armées Desgenettes, Lyon, France; ^12^Service de Médecine Intensive Réanimation, CHU Dijon, Dijon, France

**Keywords:** septic shock (MeSH), refractory septic shock, dexmedetomidine, randomized control trial (RCT), vasopressor

## Abstract

**Introduction:**

Refractory septic shock (RSS) is characterized by high vasopressor requirements, as a consequence of vasopressor resistance, which may be caused or enhanced by sympathetic hyperactivation. Experimental models and clinical trials show a reduction in vasopressor requirements and improved microcirculation compared to conventional sedation. Dexmedetomidine did not reduce mortality in clinical trials, but few septic shock patients were enrolled. This pilot trial aims to evaluate vasopressor re-sensitization with dexmedetomidine and assess the effect size, in order to design a larger trial.

**Methods:**

This is an investigator-initiated, multicenter, randomized, double-blind, placebo-controlled trial, comparing dexmedetomidine versus placebo in RSS patients with norepinephrine dose ≥0.5μg/kg/min. The primary outcome is blood pressure response to phenylephrine challenge, 6 hours after completion of a first challenge, after study treatment initiation. Secondary outcomes include feasibility and safety outcomes (bradycardia), mortality, vasopressor requirements, heart rate variability, plasma and urine catecholamines levels. The sample size is estimated at 32 patients to show a 20% improvement in blood pressure response to phenylephrine. Randomization (1:1) will be stratified by center, sedation type and presence of liver cirrhosis. Blood pressure and ECG will be continuously recorded for the first 24 h, enabling high-quality data collection for the primary and secondary endpoints. The study was approved by the ethics committee “Sud-Est VI” (2019-000726-22) and patients will be included after informed consent.

**Discussion:**

The present study will be the first randomized trial to specifically address the hemodynamic effects of dexmedetomidine in patients with septic shock. We implement a high-quality process for data acquisition and recording in the first 24 h, ensuring maximal quality for the evaluation of both efficacy and safety outcomes, as well as transparency of results. The results of the study will be used to elaborate a full-scale randomized controlled trial with mortality as primary outcome in RSS patients.

**Trial registration:**

Registered with ClinicalTrials.gov (NCT03953677). Registered 16 May 2019, https://clinicaltrials.gov/ct2/show/NCT03953677.

## Introduction

### Background and rationale

Nearly half of deaths attributable to septic shock occur in the first 3 days (1, 2), and are directly related to the consequences of circulatory failure. In these patients, the persistence of shock leading to rapid death is often termed refractory septic shock (RSS), even though there is no consensual definition for this concept. Most definitions of RSS include or are limited to a threshold of norepinephrine dosage (3–5), as RSS is characterized by persistent arterial hypotension and the need to increase catecholamines doses (5, 6). Vasoplegia is strongly and independently associated with mortality during septic shock (7).

One of the main determinants of vasoplegia during septic shock is vascular resistance to vasoactive hormones, especially catecholamines (8), as shown by altered dose-response curves to phenylephrine (9, 10). Furthermore, marked sympathetic hyperactivation has been observed during septic shock (11), with impaired cardiac and vasomotor baro-reflex and high circulating levels of endogenous catecholamines (12).

Sympatholytic substances like α-2 agonists have been tested in experimental sepsis models, and have yielded interesting results, with improvements in survival, inflammatory response, and surprisingly, blood pressure (13–15).

In parallel, a subgroup analysis of septic patients from the MENDS trial (16), comparing lorazepam to dexmedetomidine, corroborated animal models, showing not only a survival benefit with α2-agonists but also a reduction in vasopressor requirements (17). This was confirmed by a *post hoc* analysis of a small subgroup of septic shock patients from the negative SPICE III trial (18), concluding that there was a reduction in vasopressor requirements [norepinephrine/mean arterial pressure (MAP) ratio] with dexmedetomidine (19). This effect, already observed in the context of cardiac surgery (20), may appear paradoxical, since α-2 agonists are rather known for their hypotensive side-effects.

The hypothesis was then put forward that sepsis-induced downregulation of adrenoreceptors was in fact a direct consequence of sympathetic hyperactivation, and that its reversal (or “deactivation”) using α-2 agonists could restore vasopressor responsiveness (21). Experimental studies were conducted to explore this hypothesis. Clonidine and dexmedetomidine effectively restored the vasopressor response to norepinephrine to baseline levels in lipopolysaccharide-challenged rats (22). These results were confirmed in an ovine sepsis model where administration of α-2 agonists restored vascular response to vasopressors, decreased norepinephrine requirements and improved renal microcirculation (23, 24).

In 2017, a randomized controlled trial investigated the effect of dexmedetomidine in patients with sepsis. The results demonstrated reduced mortality only in the subgroup of patients with the most severe sepsis (25), and improved lactate clearance in shock patients (26). Smaller clinical trials obtained similar results (27, 28).

These clinical data support the hypothesis that the use of α-2 agonists for sympathetic deactivation may be all the more beneficial when vasopressor hyporesponsiveness is profound, as is the case in RSS.

### Objectives

This pilot trial aims to evaluate the direct effect of dexmedetomidine on vasopressor response in RSS patients, and assess the effect size and safety, in order to support the feasibility and help to design a larger trial with mortality as a primary outcome.

Our primary aim is to obtain a proof of concept of dexmedetomidine's efficacy by comparing the blood pressure response to a phenylephrine (PE) challenge between RSS patients receiving dexmedetomidine and those receiving placebo. Our secondary (albeit equally important) aims comprise safety of use, feasibility, and secondary efficacy objectives (improvement of blood pressure, vasopressor requirements, sympathetic function, organ failures, shock biomarkers, microcirculatory dysfunction, mechanical ventilation duration, and early and late mortality).

## Methods and analysis

### Trial design

**Figure 1:**
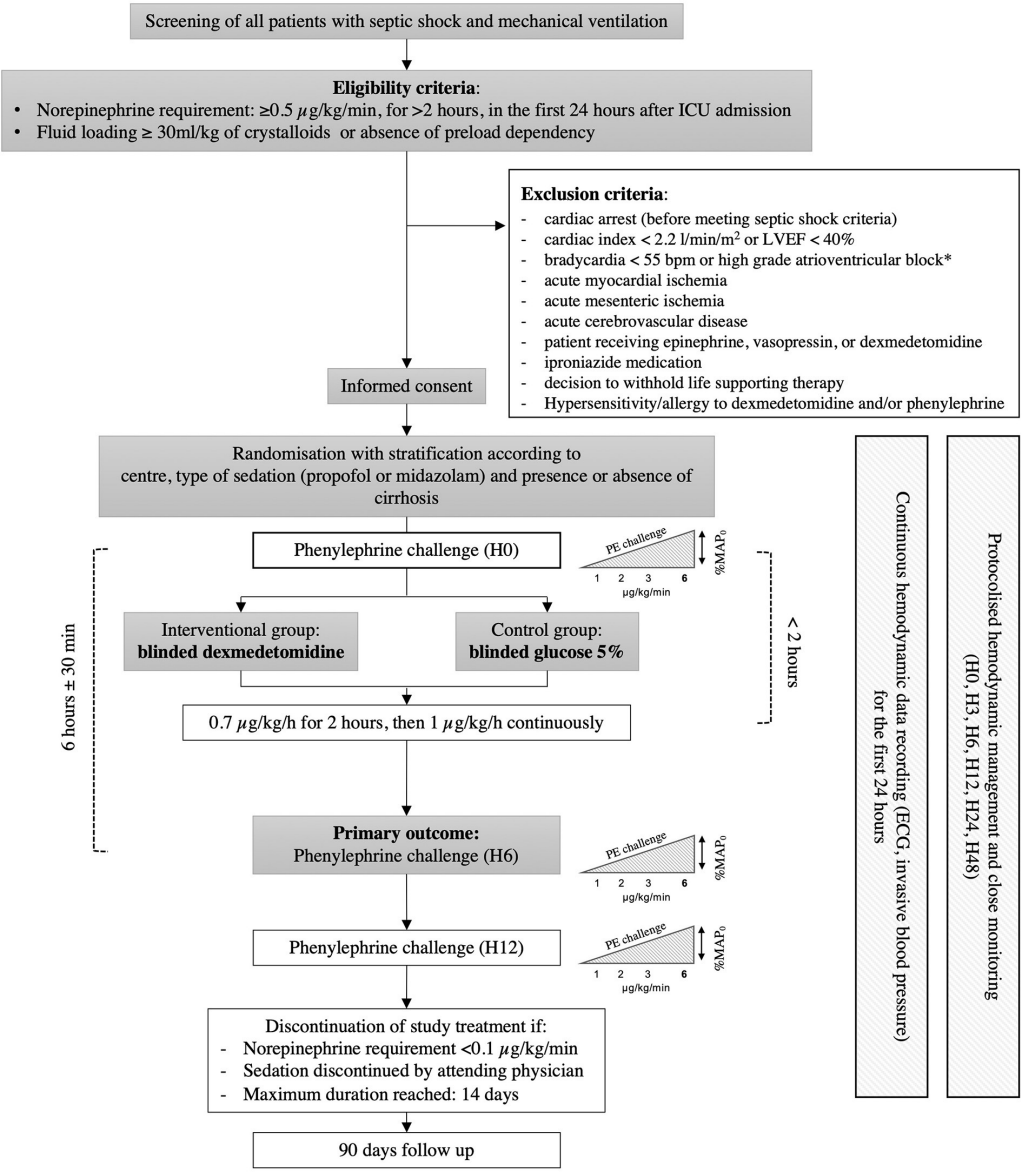
Schedule of enrollment, interventions, and assessments. ICU, intensive care unit; ECG, electrocardiogram; SAPS II, simplified acute physiology score II; SOFA, sequential organ failure assessment.

The ADRESS Pilot study is an investigator-initiated, multicenter, randomized, double-blind, placebo-controlled trial, comparing dexmedetomidine vs. placebo in RSS patients. Patients are randomly allocated (1:1 ratio) to receive either dexmedetomidine (0.7 μg/kg/h for 2 h, then 1 μg/kg/h) or placebo (glucose 5%), combined (in both arms) with protocolized fluid and vasopressor support (see below). The ADRESS Pilot study is being performed in 4 ICUs in France. A schedule of enrollment, interventions, and assessments is provided in Figure 1.

### Ethics

The study is conducted in accordance with the principles of the Declaration of Helsinki. It was approved by the central ethics committee (Ethics Committee Sud-Est VI, Clermont-Ferrand, France, 10 May 2019) with the registration number (EudraCT) 2019-000726-22. The study was also approved by the French drug safety authority (Agence Nationale de sécurité du medicament (ANSM), 29 April 2019).

### Eligibility criteria

Adult patients admitted to the ICU with refractory septic shock [Sepsis-3 criteria (29)] and requiring mechanical ventilation are considered eligible. Full inclusion and exclusion criteria are summarized in Table 1.

**Table 1:** Inclusion and exclusion criteria.

**Inclusion criteria**	**Exclusion criteria**
1. Age ≥18 years	1. Cardiac arrest before inclusion^**^
2. Septic shock, as defined by the “Sepsis-3” criteria (29)	2. Cardiac index <2.2 l/min/m^2^ OR LVEF <40%
3. Crystalloid infusion ≥30 ml/kg or absence of preload dependency^*^	3. Bradycardia <55 (unexplained by beta blockers) or high-grade atrioventricular block
4. Norepinephrine ≥0.5 μg/kg/min within 24 h after ICU admission	4. Acute myocardial ischemia (proven or suspected)
5. With persistent circulatory failure. One or more of the following criteria present within 2 h of randomization:	5. Acute mesenteric ischemia (proven or suspected)
• Arterial lactate >2 mmol/l	6. Acute cerebrovascular disease <2 weeks prior to inclusion
• Mottling score ≥1	7. Severe acute liver failure (factor V <50%)
• Oliguria (<0.5 ml/kg/h)	8. Patient already receiving epinephrine, vasopressin, or dexmedetomidine
6. Invasive mechanical ventilation	9. Iproniazide medication
7. Sedation with either propofol or midazolam	10. Decision to withhold life supporting therapy
8. Affiliation to a national health insurance scheme	11. Allergy to dexmedetomidine and/or phenylephrine
	12. Person under legal protection
	13. Pregnant or breastfeeding women

* Preload dependency is assessed by respiratory variation of inferior vena cava diameter and/or pulse pressure variations and/or passive leg raising.

** Unless septic shock criteria were met before cardiac arrest.

ICU, intensive care unit; LVEF, left ventricle ejection fraction.

### Interventions

**Figure 2:**
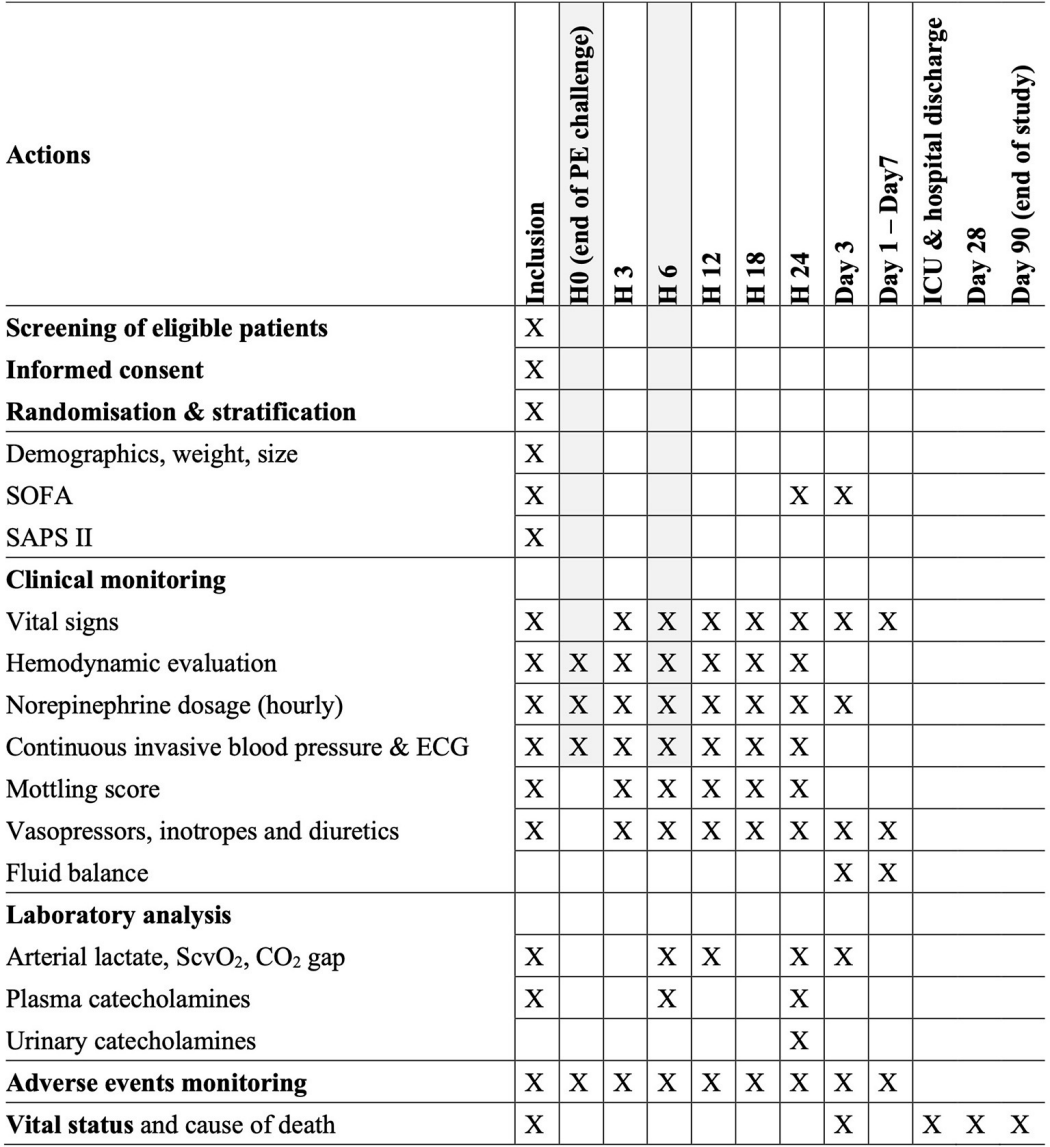
Flowchart of study procedures. *In the absence of a functioning pacemaker. LVEF, left ventricle ejection fraction.

An overview of the trial procedures is given in Figure 2.

#### Phenylephrine challenge

A phenylephrine (PE) challenge will be used to determine vasopressor responsiveness by measuring the dose-response relationship with MAP. The response will be defined as the relative change in MAP compared to baseline (before phenylephrine, MAP_0_), obtained at a given dose (MAP_d_), expressed as a percentage (% MAP_d_ = MAP_d_ / MAP_0_ × 100).

We will use the continuous perfusion protocol, already validated in several clinical studies (9, 10). PE will be infused continuously (peristaltic infusion pump) in successive dose increments at 1, 2, 3, and 6 μg/kg/min, each dose maintained for 5 min. In the study by Conrad et al. (10), the response at the 6 μg/kg/min dose level was the one that offered the best area under the curve (AUC) for distinguishing patients with RSS. Based on these findings, we chose to stop the infusion at this level to limit the duration of PE infusion.

At each dose level, the average value of each of the following parameters: systolic blood pressure, diastolic blood pressure, MAP and heart rate (HR) recorded during the last minute of infusion will be collected.

Phenylephrine will be discontinued if MAP is >140 mmHg or if bradycardia (HR <55 bpm) or ventricular arrhythmias are present. In case of thrombocytopenia with platelet count below 50,000 /mm^3^, the PE infusion will be stopped for a MAP >110 mmHg because of the increased risk of hemorrhage. The dose of norepinephrine will be maintained constant throughout the phenylephrine test.

A PE challenge will be performed immediately after enrolment, before the start of the study treatment. H0 defines the time of the first PE challenge completion. It will be repeated 6 h ± 30 min (H6) and 12 h ± 30 min (H12) after the end of the initial PE challenge.

#### Interventional group: dexmedetomidine

Patients assigned to the interventional group will receive blinded dexmedetomidine (DEXDOR^®^, 100 μg/ml, OrionPharma, Finland) in a continuous infusion (8 μg/ml dilution in glucose 5%) beginning at 0.7 μg/kg/min for 2 h before increasing to the fixed dose of 1 μg/kg/h. This dose was carefully chosen as a compromise between optimal effect and potential risk. Experimental studies showing beneficial hemodynamic effects of dexmedetomidine used high doses ranging from 1 to 100 μg/kg/h in small animals. The recommended therapeutic dose range is 0.7–1.4 μg/kg/h. We chose the intermediate dose of 1 μg/kg/h to limit the risk of hemodynamic adverse effects (hypotension, bradycardia).

#### Placebo group: Glucose 5%

Patients assigned to the placebo group will receive blinded glucose 5% in a continuous infusion using the same device (electric syringe) and label.

#### Duration of study treatment

The blinded study treatment will be initiated as soon as possible after randomization and completion of the first PE challenge. It will be maintained continuously until norepinephrine requirement decreases below 0.1 μg/kg/min, and/or sedation is discontinued. Before definitive discontinuation, the study treatment dose will be reduced by a half for 2 h.

### Outcomes

#### Primary endpoint

The primary endpoint is the MAP response to phenylephrine at H6 (6 h after the completion of the first PE challenge), expressed as the relative variation in MAP compared to baseline (before phenylephrine, MAP_0_), obtained with the maximum PE dose (MAP_dmax_) administered during PE challenge. It will be expressed as a percentage (% MAP_dmax_ = MAP_dmax_/MAP_0_x100).

#### Secondary endpoints

Secondary endpoints include the following:

#### Feasibility outcomes

Recruitment potential will be assessed, based on the screening logs in each center (proportion of recruited patients over total sedated septic shock patients). Indeed, the results of this pilot trial will inform the decisions for the design of a definitive RCT. This definitive trial will be conducted unless major safety concerns are raised by the steering committee. The protocol of this trial will be amended depending on several outcomes from the pilot trial: estimated effect-size on survival and organ dysfunction (for sample size calculation), feasibility of randomization and blinding procedures, recruitment potential (to adapt the number of centers and recruitment duration).

#### Safety outcomes

Occurrence of severe bradycardia during treatment duration, defined as heart rate <50 bpm. Hypotension is another expected side effect of dexmedetomidine. However, the opposite effect is expected as per the study hypothesis. Both blood pressure and norepinephrine doses are closely monitored during the first 24 h. Continuous blood pressure recording will make it possible to detect adverse reactions in the treatment group.

#### Efficacy outcomes

1. Course of response to PE (%MAP_0_) between H0 and H6 and between H0 and H12.
2. Cumulative and peak dose of norepinephrine at H6 and H12.
3. Mean blood pressure (systolic, diastolic and MAP) averaged from continuous recording during the first 24 h, with percentage of time spent within the MAP target, and MAP area under the curve (AUC).
4. Heart rate variability (HRV), averaged from continuous recording, at randomization, H6, H12, H24 (parameters of time and frequency domain).
5. Lactate, central venous oxygen saturation (ScvO2) and veno-arterial CO_2_ gradient at randomization and at H6, H12, H24 and daily for the first 7 days.
6. Daily SOFA score, from day 0 to day 3.
7. Cumulative fluid balance and resuscitation fluid volume, over the first 7 days.
8. Number of days without mechanical ventilation over the first 28 days (in the event of death, the patient will be considered to have been mechanically ventilated between the date of death and D28).
9. Number of days without vasopressors over the first 28 days (in the event of death, the patient will be considered to have received vasopressors between the date of death and D28).
10. Mortality at day 3, during ICU stay, and at 90 days.
11. Mottling score, at randomization, H3, H6, H12, H18, and H24.

#### Exploratory outcome

Sympathetic activity assessed by plasma and urinary levels of endogenous catecholamines. Blood samples (plasma catecholamines and methoxylated derivatives) will be taken at inclusion (before the phenylephrine challenge), H6 and H24. Collection of 24-h urine will begin before the first phenylephrine test.

### Standardized care protocol

#### Vasoactive treatments

Norepinephrine will be used in all patients. The dose of noradrenaline bi-tartrate will be reported and used for the inclusion criteria. The recommended MAP target is 65–75 mmHg for all patients. The use of vasopressin is authorized as a rescue treatment after the evaluation of the primary endpoint (H6). The use of adrenaline as a rescue treatment will be preferred in cases of impaired myocardial function. Betablockers use is prohibited.

#### Inotropes

The presence of a severe cardiogenic shock (cardiac index < 2.2 l/min/m^2^) is a non-inclusion criterion. However, patients can be recruited if cardiac output is maintained by inotropic treatment and in the absence of suspected acute coronary syndrome. Dobutamine is the preferred inotrope agent.

#### Corticosteroids

Corticosteroid replacement therapy will be prescribed, with the combination of hydrocortisone (200 mg/24 h) and fludrocortisone (50 μg tablet once a day) (30). Corticosteroid therapy will be discontinued after norepinephrine weaning.

#### Fluids

Adequate monitoring and correction of hypovolemia is of paramount importance in the management of RSS. Signs of hypovolemia will be iteratively sought at H0, H3, H6, H9, H12, H18, H24, and twice daily until the third day (or until norepinephrine weaning). One of the following methods should be used:

- Respiratory variation of the inferior vena cava (IVC) using ultrasonography >18%, or collapsed IVC (in the absence of right ventricular systolic dysfunction and in volume-controlled mode with a tidal volume ≥8 ml/ kg).
- Pulse pressure respiratory variation >13% in the absence of arrhythmia.
- Response to passive leg raising with increased stroke volume (assessed by ultrasound or with a pulse contour device) ≥15% or increase in pulsed blood pressure ≥10% (31).

In accordance with recent literature data (32), the use of balanced crystalloids is encouraged over normal saline and colloids.

#### Sedation

For basal sedation, the use of either propofol or midazolam alone is encouraged. Until evaluation of the primary endpoint, the dose of sedation will be maintained constant. The basal sedation dose will be then adjusted if necessary, to obtain a target Richmond Agitation-Sedation Scale (RASS) score. The same basal sedative drug will be used throughout the patient's stay. The dose and type of sedation will be recorded daily.

#### Renal replacement therapy (RRT)

The internationally adopted emergency criteria for starting an RRT are as follows (33): acidosis with pH <7.15 and/or hyperkalaemia > 6.5 mmol/l with EKG abnormalities. In the absence of these criteria and in light of recent data (34), we suggest postponing the initiation of RRT in patients with persistent acute kidney injury until hemodynamic stabilization is achieved.

### Randomization and allocation concealment

A computer-generated randomization will be carried out online by the investigator using the secure CleanWeb platform, after verification of the eligibility criteria. Randomization will be performed in a 1:1 ratio, stratified by center, and with minimization by sedation type (midazolam or propofol) and the presence of cirrhosis.

After randomization, the treatment arm is disclosed to the hospital's central pharmacy via a secured email. The pharmacist prepares the treatment according to the randomization arm and the patient's weight. An unmarked, sealed transportation case is used for transport to the ICU ward.

### Blinding

Preparation and blinding of the treatment syringe will be delegated to a nurse who is not in charge of the patient.

Unblinding will be mandated in the event of: occurrence of an unexplained or possibly toxic death; occurrence of a serious adverse event when knowledge of the product administered is necessary for patient care; accidental or intentional taking by a person other than the test participant.

### Data management

#### Data collection

Data will be collected on an anonymized electronic-Case Report Form (e-CRF) by a trained investigator or research assistant at each center. At inclusion, the following data will be recorded: informed consent, demographic characteristics, vital signs, SOFA and SAPS II assessment, sedation type and dosage, complete hemodynamic evaluation [norepinephrine and other catecholamines dosage, preload-dependency assessment, cardiac output measurement, clinical signs of shock including mottling score (35)].

In the first 24 h, the following data will be collected: real-time (beat-to-beat) invasive blood pressure, routine blood samples at H6, H12, H24 (± 1 h) for arterial lactate, ScvO2, veino-arterial CO2 gradient. Hemodynamic parameters will be recorded at inclusion, H3 (±1 h), H6 (±1 h), H12 (±1 h), H18 (±2 h), H24 (±2 h). Additional blood samples will be collected at inclusion, H6 and H24 for the measurement of plasma catecholamines. Patient's urine will be collected after inclusion for 24 h urinary catecholamine measurement.

During the ICU stay, the following data will be collected: daily full clinical examination, daily SOFA until day 3, continuous monitoring of vital signs, recording of organ support treatments (i.e., mechanical ventilation, RRT, catecholamines), fluid balance assessment, standard biological parameters, reporting of bradycardia episodes (heart rate <50 bpm) with associated treatments, occurrence of other adverse events.

Vital status will be assessed on day 3 (72 h after the first PE test), on discharge from ICU and from hospital, and at 28 and 90 days. If the patient has been discharged from the hospital, vital status will be obtained by calling the patient directly or by contacting their general practitioner. In the event of death, the date and cause of death will be recorded.

#### Electronic health record

Due to the continuous monitoring data needed for the primary and secondary endpoints, electronic data is recorded for each patient. Data extraction software (iCollect^®^, General Electrics Healthcare, and RECAN, α-2) will be used. Electrocardiogram and invasive blood pressure curve will be continuously extracted (500 Hz) for 24 h and saved with time stamps to allow high quality (e.g., averaging invasive blood pressure) and blinded analysis of PE challenge. This also makes it possible to compute for each patient the time spent above/below the target MAP, and to compute HRV analysis from continuous ECG.

#### Data monitoring

The trial is overseen by an independent steering committee. Meetings are scheduled on a regular basis until the end of enrolment period. Research assistants from the coordinating Centre will regularly monitor all the centers on site to check adherence to the protocol and the accuracy of the recorded data.

### Statistical methods

#### Sample size estimation

Mean blood pressure response to PE at 6 μg/kg/min in RSS patients was reported to be 122% ± 25%, compared to 163% in patients without RSS (10). We hypothesize that the addition of dexmedetomidine will result in an average response of 147% ± 25%, an improvement of 20%. Under these assumptions with a 5% alpha risk and 80% power, 16 analyzable patients per group are required. To account for non-analyzable patients, 36 patients (18 per group) will be included. Randomization will be stratified on the type of associated sedation and of the presence of liver cirrhosis, as dexmedetomidine efficacy may vary according to the midazolam or propofol use, and to the autonomous nervous system activity impairment caused by liver cirrhosis.

#### Statistical analysis

All the analyses will be performed using SAS version 9.4 (SAS Institute Inc., Cary, NC). A two- tailed *p* < 0.05 will be considered as indicating statistical significance.

Categorical variables will be presented as number and percentage and continuous variables as mean ± standard deviation (SD) or median [interquartile range (IQR)], according to their distribution. Characteristics will be compared between groups by the chi square or Fisher's exact tests, or the Student t or Mann-Whitney U tests, as appropriate.

The analysis will be performed on an intention-to-treat (ITT) basis. In addition, a modified ITT (mITT; excluding wrongly included patients), and per protocol analyses will be performed. Sensitivity analysis will be performed using multivariate linear regression, with the primary endpoint as outcome and the treatment group as the main explanatory variable, adjusted for randomization factors (Centre, sedation treatment and cirrhosis).

Secondary criteria will be analyzed with the Emax model with random effects, usually used in the analysis of dose-response curves with repeated measures (9, 10), to describe the relationship between %MAP_d_ (= MAP_d_ / MAP_0_) and phenylephrine doses in the two groups, at baseline, H6 and H12. Dose-response curves will be compared between the two treatment groups, according to the Emax and ED50 parameters measuring, respectively, the maximum effect on %MAP_d_ and the growth speed (i.e., the PE dose in which half of Emax is produced).

### Recruitment

Recruitment started in October 2019. Recruitment was suspended at the beginning of the COVID-19 pandemic, from March 2020 to January 2021. A protocol amendment enabling an 18-month prolongation of the inclusion period has been approved. Recruitment has resumed and is currently ongoing.

## Discussion

The present study will be the first randomized trial to specifically address the hemodynamic effects of dexmedetomidine in patients with refractory septic shock.

We chose to target a population of patients with RSS, at high risk of death. Indeed, although sympathetic hyperactivation is present in most septic shock patients, catecholamine levels are significantly higher in non-survivors (12). Thereby, RSS patients are probably the most likely to benefit from the sympathetic deactivation strategy with dexmedetomidine to improve their outcome. The efficacy of α-2 agonists specifically in RSS has been reported, but only in case reports so far (36, 37). We chose dexmedetomidine over clonidine because it has many advantages: it is approved for sedation of critically ill patients, and has a short half-life without alteration in patients with impaired renal function (38).

Despite its potential efficacy, the safety of use of dexmedetomidine in this population is an open question, and is among the main outcomes of this pilot trial. Intensivists may be reluctant to use dexmedetomidine in the context of shock, due to the usual effects of this drug such as hypotension and bradycardia. However, blood pressure was improved by dexmedetomidine in septic hypotensive patients dependent on vasopressors (25, 39). Bradycardia is present in 10% of critically ill patients (requiring intervention in only 5%) (39), but it was not significantly increased in a study including patients with septic shock (25). Furthermore, no asystole, cardiac arrest or death were reported in clinical trials.

One of the strengths of this trial is to promote the personalization of the management of a well-defined subgroup of patients, according to pathophysiological characteristics. Furthermore, we implemented a high-quality process for data acquisition and recording in the first 24 h. This will allow maximal quality for the evaluation of both efficacy (pressure response curves to PE, HRV analysis, achievement of target MAP), and safety (bradycardia) outcomes, as well as transparency of results.

Nevertheless, our study also has some limitations. First, the study procedures involving a PE challenge, hemodynamic evaluations, and data recording will be time-consuming and might restrain inclusions outside of the working hours, which may bias the evaluation of the recruitment potential for the definitive trial. Thus, we may consider that recruitment rate could probably be higher in the future trial, without PE challenges. Second, the blinding procedure is not optimal, because the study drug is prepared by an unblinded nurse. Preparation in the hospital's pharmacy could have overcome this potential bias, but it would add a significant delay in the administration of the drug, and restrain inclusions outside of the working hours. Finally, one of the main limitations for the interpretation of results will be the small sample size of the study, especially in regard of the multiple outcomes we plan to analyze. This is due to the “pilot,” exploratory nature of the study. The risk of bias generated by multiple analyses can be compensated by a careful physiologic approach: a precise effect of the drug is researched using predefined criteria in a selected population, controlling for the mechanistic pathways involved (autonomous nervous system activity, catecholamines dosages).

We believe that a preliminary pilot study was necessary in this case, for several reasons: (1) to confirm the safety of this drug in this particular context; (2) to assess the feasibility and recruitment potential; and (3) to confirm the vasopressor-enhancement effect of the drug in this specific population of patients, which would justify its use. This latter point was taken as the primary objective, since the reality of this drug's effect is still debated, notably because the effect in the patients targeted in our trial diverges from the effect observed in non-shock patients. Pilot trials are not meant to draw conclusions on an intervention's efficacy. Here, we rather chose a surrogate of the drug's effect (i.e., vasopressor response), to indicate its potential effectiveness. The primary clinical endpoint of the definitive RCT will likely be survival.

## Ethics statement

The study was approved by the Central Ethics Committee (Ethics Committee Sud-Est VI, Clermont-Ferrand, France, 10 May 2019) with the registration number (EudraCT) 2019-000726-22. The patients/participants provided their written informed consent to participate in this study.

## Author contributions

AD, J-PQ, and LQ conceived and designed the study. IF, AB, BL, LA, JB, and AC contributed to the design. AD and J-PQ drafted and revised the manuscript. All authors contributed to the article and approved the submitted version.

## Funding

The ADRESS Pilot study is an investigator-initiated trial supported by public funds (GIRCI grant) obtained in 2018.

## Conflict of interest

The pharmaceutical company ORION Pharma, manufacturer of DEXDOR®, agreed to provide the necessary amount of drug to conduct the study. ORION Pharma did not provide financial support, or any other form of support. The authors declare that the research was conducted in the absence of any commercial or financial relationships that could be construed as a potential conflict of interest.

## Publisher's note

All claims expressed in this article are solely those of the authors and do not necessarily represent those of their affiliated organizations, or those of the publisher, the editors and the reviewers. Any product that may be evaluated in this article, or claim that may be made by its manufacturer, is not guaranteed or endorsed by the publisher.
